# ﻿Three new species of Polleniidae (Diptera) from China

**DOI:** 10.3897/zookeys.1228.129419

**Published:** 2025-02-18

**Authors:** Shixin Liu, Honghu Ji, Wenliang Li, Gang Yao

**Affiliations:** 1 College of Horticulture and Plant Protection, Henan University of Science and Technology, Luoyang, Henan 471023, China Henan University of Science and Technology Luoyang China; 2 Jinhua Academy of Agricultural Sciences, Jinhua 321000, China Jinhua Academy of Agricultural Sciences Jinhua China; 3 College of Agriculture, Jinhua University of Vocational Technology, Jinhua 321007, China Jinhua University of Vocational Technology Jinhua China

**Keywords:** Calyptratae, cluster flies, *Dexopollenia*, identification key, Oestroidea, taxo­nomy, *Xanthotryxus*

## Abstract

*Dexopolleniaguangxiensis* Liu, Li & Yao, **sp. nov.**, *Dexopolleniachongqingensis* Liu, Li & Yao, **sp. nov.** and *Xanthotryxushuapingensis* Liu, Li & Yao, **sp. nov.** from the Guangxi Zhuang Autonomous Region and Chongqing are described and illustrated. Keys to species of the genera *Dexopollenia* and *Xanthotryxus* from China are provided. Photographs of the habitus and male genitalia of the new species are also provided.

## ﻿Introduction

The genus *Pollenia* Robineau-Desvoidy, 1830 was erected for the type species *Muscarudis* Fabricius, 1794, the earliest recorded cluster fly. The Polleniidae family group name was originally proposed by Brauer and Bergenstamm (1889) to include the single genus *Pollenia* (Gisondi et al. 2020). Later, *Pollenia*, *Dexopollenia* Townsend, 1917, *Xanthotryxus* Aldrich, 1930 and *Anthracomyia* Malloch, 1927 were treated as composing the subfamily Polleniinae (or tribe Polleniini) in Calliphoridae (Hall 1965; Dear 1986; Schumann 1986; Kurahashi 1989; Gisondi et al. 2020). The taxonomic status of Polleniinae has been debated. Over the past few years, molecular data consistently retrieved Polleniidae as sister to Tachinidae and phylogenetically distant from the ‘core’ Calliphoridae, but this sister-group relationship has remained practically without support from morphological evidence (Singh and Wells 2013; Kutty et al. 2019; Gisondi et al. 2020). Until the study of Cerretti et al. (2019), molecular-based phylogenetic analyses retrieved members of the former calliphorid subfamily Polleniinae as sister to Tachinidae; also, this clade is consistently reconstructed as distinct from the non-polleniine calliphorid clades with strong statistical support. On the other hand, a sister-group relationship between the former calliphorid subfamily Polleniinae and Tachinidae is supported by one non-homoplastic synapomorphy: ventral and ventrolateral surface of distalmost parts of the distiphallus smooth. All existing evidence shows that Polleniidae is monophyletic. Johnston et al. (2023) found in their study of subfamilial phylogenetic relationships within Polleniidae, that *Pollenia* forms a monophyletic clade, which is sister to the genera *Melanodexia-Morrinia-Dexopollenia*, and recovered *Dexopollenia-Xanthotryxus* Aldrich as sister to the remaining Polleniidae comprising *Pollenia* and sister-group *Morinia-Melanodexia*. Gisondi et al. (2023) studied the phylogenetic relationships within Polleniidae and obtained a morphological topology that is largely consistent with the findings of Johnston et al. (2023), particularly, that *Pollenia* is the sister taxon of the *Morinia-Melanodexia* clade, and suggested that Polleniidae be divided into Moriniinae and Polleniinae.

The family Polleniidae may be diagnosed as follows: Small to medium-sized oestroid flies varying from yellow to black in ground colour. Stem vein bare dorsally. Anal vein not reaching wing margin. Posterodorsal margin of hind coxa bare. Prosternum and proepisternal depression bare. Female: ovipositor sclerite length moderate; sternite 8 of ovipositor elongate with apex entire; cerci long and narrow. Male: ventral and ventrolateral surface of distalmost parts of distiphallus smooth (Cerretti et al. 2019).

Polleniidae accounts for around 150 described species in eight genera worldwide (Cerretti et al. 2019; Gisondi et al. 2020; Xue et al. 2020; Gisondi et al. 2023). Five of the eight genera (and 30 species) are known from China (Fan 1992; Fan 1997; Xue and Zhao 1996; Xue et al. 2020; Xue and Du 2022). *Dexopollenia* comprises 21 species (Cerretti et al. 2019; Gisondi et al. 2020; Xue and Du 2022), and the following nine species are known to occur in China: *D.aurantifulva* Feng, 2004; *D.disemura* Fan & Deng, 1993; *D.flava* Aldrich, 1930; *D.geniculata* Malloch, 1935; *D.luteola* Villeneuve, 1927; *D.maculata* Villeneuve, 1933; *D.nigriscens* Fan, 1992; *D.tianmushanensis* Fan, 1997; and *D.uniseta* Fan, 1992. *Xanthotryxus* comprises seven species, all distributed in China (Gisondi et al. 2020; Xue et al. 2020): *X.aurata* Séguy, 1934; *X.bazini* Séguy, 1934; *X.draco* Aldrich, 1930; *X.ludingensis* Fan, 1992; *X.melanurus* Fan, 1992; *X.mongol* Aldrich, 1930; and *X.uniapicalis* Fan, 1992. *Dexpollenia* and *Xanthotryxus* are distinct from West Palaearctic *Pollenia*, but preimaginal stages of *Dexopollenia* and *Xanthotryxus* are almost unknown in comparison to West Palaearctic Polleniidae (Kurahashi 1967; Szpila 2003; Grzywacz et al. 2012; Szpila et al. 2023).

In this article, two new species belonging to *Dexopollenia* and one new species belonging to *Xanthotryxus* are described, and updated keys of the two genera are provided.

## ﻿Material and methods

The specimens were photographed under a Canon EOS 5DsR camera (Tokyo, Japan) with a Laowa FF 100 mm F2.8 CA-Dreamer Macro 2× lens (Hefei, China) and stacked with Helicon Focus ver. 7 software. The male genitalia were photographed under a Canon EOS 5DsR camera (Tokyo, Japan) with Mitutoyo, M Plan Apo 10× (Japan). Photographs were edited with Adobe Photoshop CC 2017. Morphological terminology mainly follows Cumming and Wood (2017) and McAlpine (1981).

Abbreviations used are as follows: **acr**—acrostichal setae; **dc**—dorsocentral setae; **ial**—intra-alar setae; **h**—humeral setae; **ph**—posthumeral setae; **pra**—prealar setae; **sal**—supraalar setae; **pal**—postalar setae; **mpl**—mesopleural setae; **spl**—sternopleural setae; **ppl**—pteropleural setae; **a**—anterior setae; **v**—ventral setae; **d**—dorsal setae; **p**—posterior setae; **ad**—anterodorsal setae; **pd**—posterodorsal setae; **av**—anteroventral setae; **pv**—posteroventral setae; **r-m**—radio-medial cross-vein; **2R_5_**—distal fifth radial cell; **T**—tergite; **ST**—sternite.

Depositories cited in this work are as follows:

**HAUST**Insect Collection of Henan University of Science and Technology, Luoyang, Henan, China. Curator: Wenliang Li

## ﻿Results

### ﻿Taxonomy

#### ﻿Genus Dexopollenia

Taxon classificationAnimaliaDipteraPolleniidae

Townsend, 1917

9552CD03-ED74-55D5-AA8C-1314EFA4CA68

Dexopollenia Townsend, 1917: 201. Type species: Dexopolleniatestacea Townsend, 1917 (original designation).

##### Diagnosis.

Adults of this genus can be recognized by the combination of the following characters: body length 5–10 mm; eyes with hairs or bare; males frons narrow, interfrontalia surface disappears at narrowest part parafacial bare, arista plumose, vibrissa approaches epistoma; thorax with golden tomentum or soft hairs, propleura and basisternum of prothorax bare; postalar declivity with hairs, spl 1+1; subcostal sclerite with yellow hairs but no black setulae; radial stem vein and subalar knob bare, 2R_5_ open, M_1+2_ tip gently curved forward in a wide obtuse angle or very gently in an arc, lower calypter bare; T_3–5_ with strong marginal bristles on the backside (Fan 1997).

##### Distribution.

Australasian, Oriental and Palaearctic.

### ﻿Key to Chinese species of genus *Dexopollenia* Townsend, 1917 (males)

Modified from Fan (1997).

**Table:** 

1	Leg mostly yellow (Figs 3, 13), palpus yellow (Fig. 14)	**2**
–	Leg black, palpus black	**9**
2	Discal scutellar setae present	**3**
–	Discal scutellar setae absent	**5**
3	Thorax black (Figs 1, 11), tarsus yellow (Fig. 13)	**4**
–	Thorax mostly yellow, tarsus dark yellow	***D.aurantifulva***
4	Interfrontalia reddish-brown (Fig. 14), T_5_ black (Fig. 12), ST_1_ hairs black, mid tibia 1 pd	***D.chongqingensis* Liu, Li & Yao, sp. nov.**
–	Interfrontalia orange (Fig. 4), T_5_ mostly yellow (Fig. 2), ST_1_ hairs yellow, mid tibia 2 pd	***D.tianmushanensis***
5	Femur end and tibia base black	**6**
–	Femur end and tibia base yellow	**7**
6	T_3–5_ mostly black, except for a narrow yellow trailing edge band at the end	***D.geniculata***
–	T_1–5_ mostly yellow (Fig. 2), except for dark spots in the middle	***D.maculata***
7	Tarsus black (Fig. 3), ial 0+1	**8**
–	Tarsus end brown, rest yellow, ial 0+2	***D.flava***
8	Thorax yellow, mpl 0+2, abdomen yellow only, T_5_ brown	***D.uniseta***
–	Thorax black (Fig. 1), mpl 0+3, tergites except T_5_ with black trailing edge band, T_3–5_ with a black ovate spot medially (Fig. 2)	***D.guangxiensis* Liu, Li & Yao, sp. nov.**
9	Presutural acr 1	***D.disemura***
–	Presutural acr 0	**10**
10	Without facial carina, ph 1	***D.nigriscens***
–	Facial carina developed, ph 2	***D.luteola***

#### ﻿ Dexopollenia guangxiensis

Taxon classificationAnimaliaDipteraPolleniidae

Liu, Li & Yao sp. nov.

60FDC2C1-25A9-5B1B-A0A3-D29F21A1494C

https://zoobank.org/C184C0F4-76C0-4E66-980E-394BF9AAB9C3

Figs 1–5 , 6–10

##### Type material.

***Holotype*** • (dissected), male (HAUST), China: Guangxi Zhuang Autonomous Region, Guilin City, Lingui District, Huangsha Yao Township, Anjiangping, 25°55'6"N, 109°94'4"E, 1. VI. 2023, 1340 m, leg. Shixin Liu. ***Paratype*** • 1 male (HAUST), same data as holotype.

##### Diagnosis.

Eyes with sparse short hairs; first and second antennal segments dark yellow, third antennal segment black except base dark yellow; facial carina not particularly developed; eyes 2 times higher than gena; thorax black, with dense white pollen; acr 1+2, ial 0+1, ppl absent; legs yellow except tarsus black; subcostal sclerite yellowish, bare; radial stem vein and subalar knob bare; abdomen with a mediodorsal dark vitta interrupted mediodorsally; cercus broad, terminal tip in dorsal view; paraphallus tip curved forward; hypophallus and acrophallus membranous.

##### Description.

**Male.** Thorax appears black in ground colour, slightly white pollinose. Wing brownish-yellow. Legs yellow, tarsus black. Abdomen largely yellow, with a mediodorsal dark vitta interrupted mediodorsally.

**Figures 1–5.**
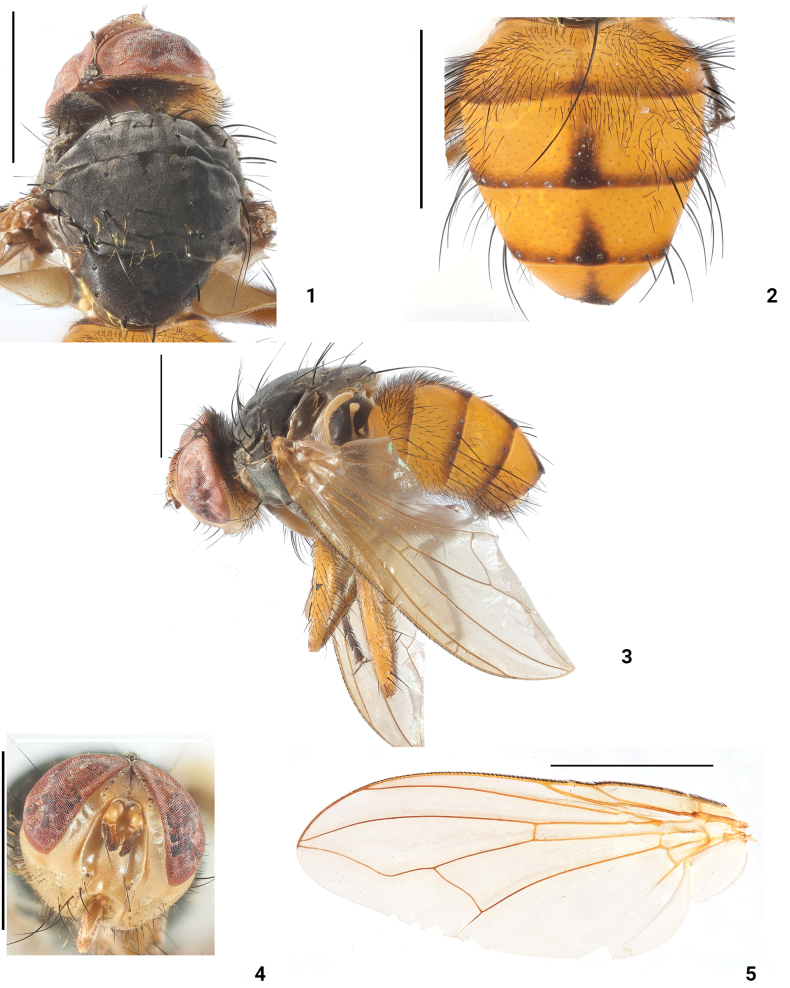
*Dexopolleniaguangxiensis* Liu, Li & Yao, sp. nov. male (holotype) **1** head, thorax, dorsal view **2** abdomen, dorsal view **3** habitus, lateral view **4** head, anterior view **5** wing. Scale bars: 3 mm.

***Head*** (Figs 1, 3, 4). Eyes red, with sparse short hairs; post-ocellar setae and post-vertical setae present, eyes holoptic; Frons narrow, interfrontalia surface disappears at narrowest part; parafrontal yellow, with six pairs of frontal setae; parafacial and ocellar triangle dark yellow, with yellowish-white tomentum; gena yellow, with dense black setae; lunule bare; first and second antennal segments dark yellow, third antennal segment black except base dark yellow, first and second antennal segments with black setae, third antennal segment about 2 times longer than second antennal segment, arista plumose, arista longer than third antennal segment, but not more than epistome; mid-facial plate dark yellow, facial carina not particularly developed; palpus yellow; eyes 2 times higher than gena; postgena concolorous with gena, all yellow; hairs on postgena mostly black, yellowish hairs posteriorly.

***Thorax*** (Figs 1, 3) black, with slightly white pruinescence; prodorsum and dorsum of mesothorax with crinkly golden hair; acr 1+2, dc 2+3, ial 0+1, h 2, ph 1+0, pra 1, sal 1, pal 2; scutellum black, with a tuft of crinkly golden hairs; anterior and posterior spiracles yellow, proepimeral setae present; anepisternum black, with crinkly golden hairs, mpl 0+3, spl 1+1; ppl absent, with a tuft of crinkly golden hairs; inferior laterotergite bare.

***Wings*** (Fig. 5) brownish-hyaline; epaulet and basicostal scale dark yellow, subcostal sclerite yellowish, bare; radial stem vein and subalar knob bare; 2R_5_ open, width 1/2 the length of r-m; upper calypter and lower calypter dark yellow, halter yellow.

***Legs*** (Fig. 3) yellow except tarsus black, fore femur with 10 pv, 4 pd; fore tibia with 3 ad, 2 pd; mid femur with 2 a; mid tibia with 2 ad, 2 p, 1 d, 2 pd; hind femora with 8 ad, 4 pd; hind tibia with 3 ad, 3 pd.

**Figures 6–10.**
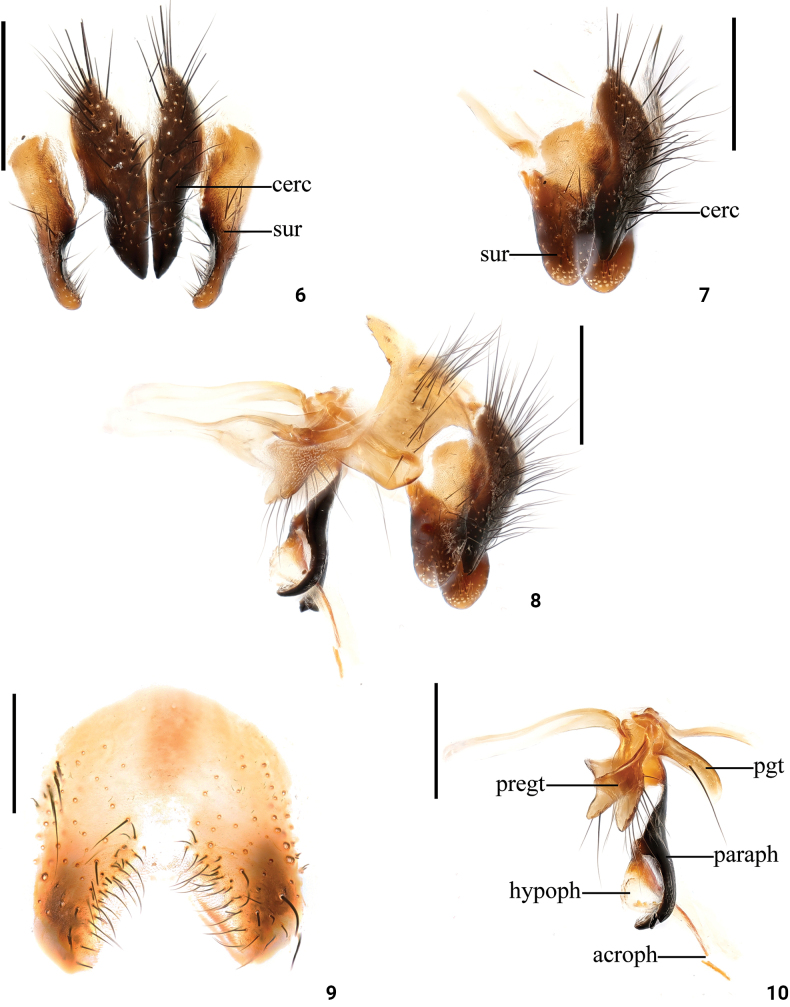
*Dexopolleniaguangxiensis* Liu, Li & Yao, sp. nov. male terminalia **6** cercus and surstyli, posterior view **7** cercus and surstyli, lateral view **8** terminalia lateral view **9**ST_5_ ventral view **10** phallic complex, lateral view. Scale bars: 0.3 mm. Abbreviations: acroph: acrophallus; cerc: cercus; hypoph: hypophallus; paraph: paraphallus; pgt: postgonites; pregt: pregonites; sur: surstyli.

***Abdomen*** (Figs 2, 3) largely yellow, with a mediodorsal dark vitta interrupted mediodorsally; ST_1–4_ with long black setae, terminal margin of lateral lobe of ST_5_ with a black spot (Fig. 9); epandrium with long dense black setae. Male terminalia: medial side of surstyli with dense black hairs in posterior view (Fig. 6); cerci broad, terminal tip in posterior view (Fig. 6); surstyli broad and terminal rounded in lateral view (Figs 7, 8); pregonite with a row of black setae, and postgonites with one black seta; paraphallus terminal curved forward; hypophallus and acrophallus membranous (Figs 8, 10).

**Female.** Unknown.

##### Measurements.

Male. Body length 5.1–8.6 mm.

##### Etymology.

The specific epithet is chosen after Guangxi Province where the holotype was collected.

##### Distribution.

China (Guangxi).

##### Remarks.

This new species is similar to *D.maculata* Villeneuve, 1933, but differs in the following points: ial 0+1, ad 2, T_1–4_ with a mediodorsal dark vitta interrupted mediodorsally, and cerci broad in dorsal view. Further, the new species paraphallus is slightly wider and rounded terminally compared to the *D.maculata* paraphallus, and the new species postgonites is longer than the *D.maculata* postgonites.

#### ﻿ Dexopollenia chongqingensis

Taxon classificationAnimaliaDipteraPolleniidae

Liu, Li & Yao sp. nov.

E05A48DB-614C-5BA6-8F99-C16CBEFA9B55

https://zoobank.org/79D82223-F59A-407F-8AEC-EC05BE4350A1

Figs 11–15 , 16–20

##### Type material.

***Holotype*** • (dissected), male (HAUST), CHINA: Red flag guard station, Yintiaoling, Wuxi County, Chongqing, 31°30'32.2972"N, 109°49'10.8334"E, 16. VIII. 2023, 1125 m, leg. Xulong Chen. ***Paratypes*** • 2 males (HAUST), same data as holotype.

##### Diagnosis.

Eyes bare; interfrontalia reddish-brown; parafrontal gray, with seven pairs of frontal setae; parafacial and ocellar triangle reddish-brown, parafacial base gray; first and second antennal segments brown, third antennal segment reddish-brown except base brown, third antennal segment about 2.5 times longer than second antennal segment, arista plumose; eyes 3 times height of gena. Thorax black, acr 1+2, dc 2+3, ial 0+2, h 2, ph 1+0, pra 1, sal 1, pal 2; scutellum dark reddish-brown. T_1+2_ all yellow, T_3–4_ with a dark stripe in the middle, T_4_ almost black, T_5_ all black; surstyli end bends to both sides in posterior view; cercus slender, terminal tip bottom 2/5 black in dorsal view; surstyli broad in lateral view.

##### Description.

**Male.** Thorax appears black in ground colour, slightly white pollinose. Wing brownish-yellow. Legs yellow. Abdomen largely yellow, T_1+2_ all yellow, T_3–4_ with a dark stripe in the middle, T_4_ almost black, T_5_ all black.

**Figures 11–15.**
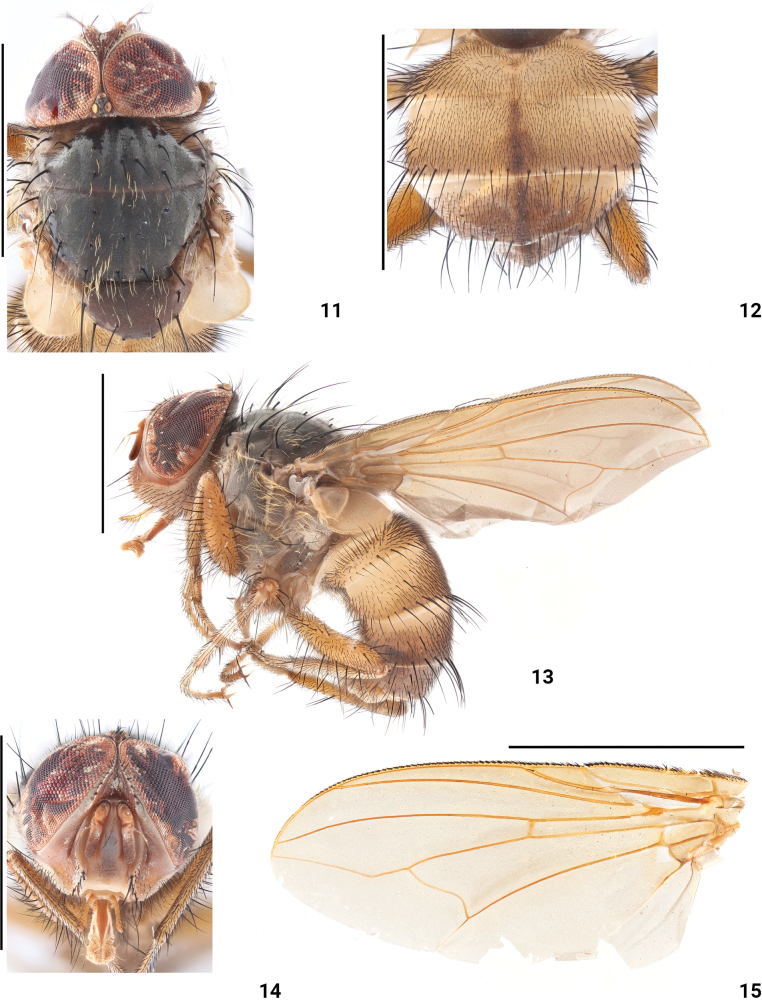
*Dexopolleniachongqingensis* Liu, Li & Yao, sp. nov. male (holotype) **11** head, thorax, dorsal view **12** abdomen, dorsal view **13** habitus, lateral view **14** head, anterior view **15** wing. Scale bars: 3 mm.

***Head*** (Figs 11, 13, 14). Eyes red, bare; eyes holoptic; frons narrow, interfrontalia surface disappears at narrowest part, interfrontalia reddish-brown; parafrontal gray, with seven pairs of frontal setae; parafacial and ocellar triangle reddish-brown, parafacial base gray; gena yellow, with dense black setae; lunule bare; first and second antennal segments brown, third antennal segment reddish-brown except base brown, third antennal segment about 2.5 times longer than second antennal segment, arista plumose; mid-facial plate dark reddish-brown, facial carina not particularly developed; palpus yellow; eyes 3 times higher than gena; postgena concolorous with gena, all yellow.

**Figures 16–20.**
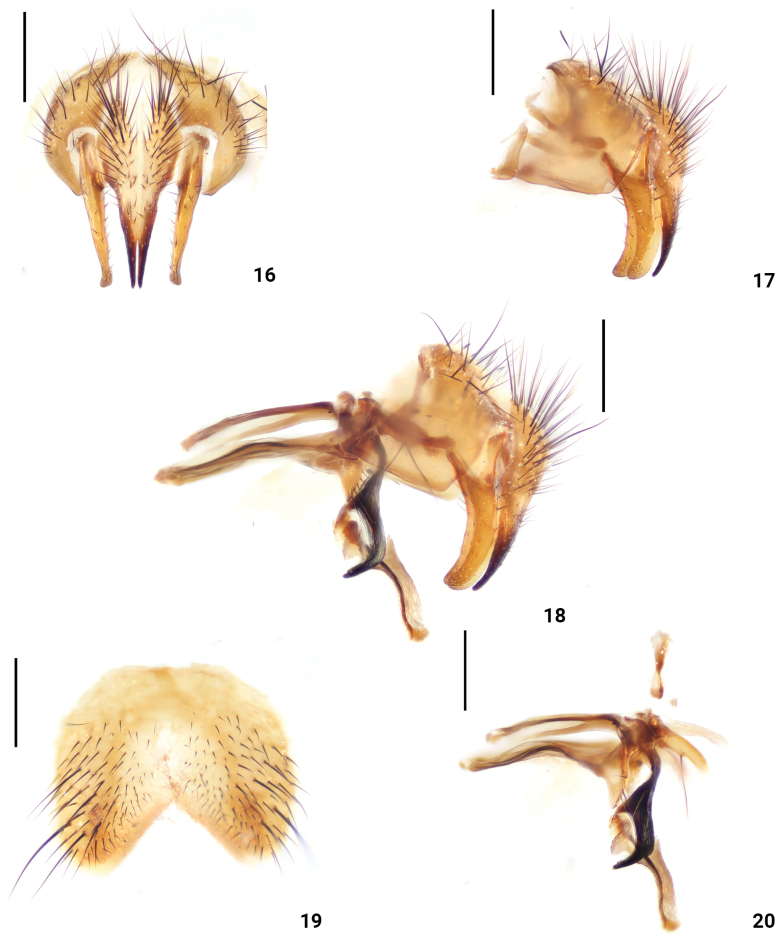
*Dexopolleniachongqingensis* Liu, Li & Yao, sp. nov. male terminalia **16** cercus and surstyli, posterior view **17** cercus and surstyli, lateral view **18** terminalia lateral view **19**ST_5_ ventral view **20** phallic complex, lateral view. Scale bars: 0.3 mm.

***Thorax*** (Figs 11, 13) black, with slightly white pruinescence; prodorsum and dorsum of mesothorax with crinkly golden hair; acr 1+2, dc 2+3, ial 0+2, h 2, ph 1+0, pra 1, sal 1, pal 2; scutellum dark reddish-brown; anterior and posterior spiracles yellow, proepimeral setae present; anepisternum black, with crinkly golden hair, mpl 0+4, spl 1+1; ppl absent, with a tuft of crinkly golden hair; inferior laterotergite bare.

***Wing*** (Fig. 15) brownish-hyaline; epaulet and basicostal scale yellow, subcostal sclerite yellowish, bare; radial stem vein and subalar knob bare; 2R_5_ open; upper calypter and lower calypter brown, halter yellow.

***Legs*** (Fig. 13) yellow, fore femur with 10 pv, 4 pd, 3 p; fore tibia with 3 p, 2 ad; mid femur with 3 pv, 2 av; mid tibia with 1 ad, 1 pd; hind femora with 7 ad, 7 av, 5 pd; hind tibia with 3 ad, 2 pd.

***Abdomen*** (Figs 12, 13) largely yellow, with dense black hairs; T_1+2_ all yellow, T_3–4_ with a dark stripe in the middle, T_4_ almost black, T_5_ all black; ST_1–4_ with long black seta, ST_5_ tip with long black seta (Fig. 19). Male terminalia: surstyli end bends to both sides in posterior view (Fig. 16); cercus slender, terminal tip bottom 2/5 black in posterior view (Fig. 16); surstyli broad in lateral view (Figs 17, 18); pregonite with a row of black seta anteriorly, and one black seta posteriorly; paraphallus terminal curved forward; hypophallus and acrophallus membranous (Figs 18, 20).

**Female.** Unknown.

##### Measurements.

Male. Body length 3.4–8.6 mm.

##### Etymology.

The specific epithet is chosen after Chongqing where the holotype was collected.

##### Distribution.

China (Chongqing).

##### Remarks.

This new species is similar to *D.maculata* Villeneuve, 1933, differing in the following points: tarsus yellow, T_4_ mostly black, and T_5_ all black. The cerci are slender and the basal 2/5 is black in posterior view. Surstyli are more slender than *D.maculata* in lateral view. The terminal paraphallus in the new species is approximately angular and curved; in *D.maculata* the terminal paraphallus is arc-shaped and bent. The lower part of the paraphallus in the new species is wide and in *D.maculata* it is slender.

#### ﻿Genus Xanthotryxus

Taxon classificationAnimaliaDipteraPolleniidae

Aldrich, 1930

894C659F-7FBD-5EEF-92B3-FE45C75345BB

Xanthotryxus Aldrich, 1930: 3. Type species: Xanthotryxusmongol Aldrich, 1930 (original designation).

##### Diagnosis.

Adults of this genus can be recognized by the combination of the following characters: bulk size, body length 9–13 mm, body totally black; eyes bare; parafacial bare or with hairs, facial carina broadly flat or slightly round, not angular; antennae black, arista plumose, vibrissa far from epistoma; scutum, scutellum and tergite with crinkly golden hairs; propleura and basisternum bare, suprasquamal ridge bare, postalar declivity with tomentum; basal tubercle of R_4+5_ with small black setae, subcostal sclerite with a tuft of setae and yellow villi; legs black; abdomen with golden tomentum, cerci slender, acrophallus well developed and hypophallus not well developed (Fan 1997).

##### Distribution.

Palaearctic, Oriental.

### ﻿Key to Chinese species of genus *Xanthotryxus* Aldrich, 1930 (males)

Modified from Fan (1997).

**Table:** 

1	Discal scutellar setae present, ST_1_ hairs all yellow or partially yellow	**2**
–	Discal scutellar setae absent, ST_1_ hairs all black	***X.bazini***
2	Presutural acr 1 or 0	**3**
–	Presutural acr 2	**4**
3	Presutural acr 1, parafacial with hairs, 2R_5_ open (Fig. 25)	***X.aurata***
–	Presutural acr 0, parafacialia bare, 2R_5_ closed	***X.ludingensis***
4	2R_5_ open (Fig. 25), opening length approximately equal to the length of r-m	**5**
–	2R_5_ narrow opening, opening length approximately 1/3 of the length of r-m	***X.melanurus***
5	Apex of cerci separated in posterior view	**6**
–	Apex of cerci united in posterior view	***X.uniapicalis***
6	Parafacialia bare (Fig. 24), h 4, ppl 3	***X.huapingensis* Liu, Li & Yao, sp. nov.**
–	Parafacialia with hairs, h 3, ppl 2	**7**
7	Frontal setae 15, T_2–5_ hairs all black	***X.mongol***
–	Frontals setae 10, T_2–5_ hairs partially yellow	***X.draco***

#### ﻿ Xanthotryxus huapingensis

Taxon classificationAnimaliaDipteraPolleniidae

Liu, Li & Yao sp. nov.

64883528-05D0-5AC6-A29F-45218BF87636

https://zoobank.org/9FC00353-209C-4EF0-833F-C29BF84B410C

Figs 21–25 , 26–30

##### Type material.

***Holotype*** • (dissected), male (HAUST), CHINA: Guangxi Zhuang Autonomous Region, Guilin City, Lingui District, Huangsha Yao Township, Anjiangping, 25°55'6"N, 109°94'4"E, 1. VI. 2023, 1340 m, leg. Shixin Liu. ***Paratypes*** • 2 males (HAUST), same data as holotype.

##### Diagnosis.

Eyes bare; third antennal segment about 2.5 times longer than second antennal segment, third antennal segment as long as distance from vibrissa to epistoma; eyes 3 times higher than gena; palpus black; h 4, ppl 3, with a tuft of crinkly golden hairs; postalar declivity with a dense tuft of crinkly golden hairs; legs black; subcostal sclerite yellowish, with dense yellow tomentum and 2–3 setae; surstyli terminal extension and hook-like in lateral view.

##### Description.

**Male.** Black species. Thorax black with crinkly golden hairs. Wing brownish-yellow. Legs black. Abdomen black tessellate yellow sarcophagids markings.

**Figures 21–25.**
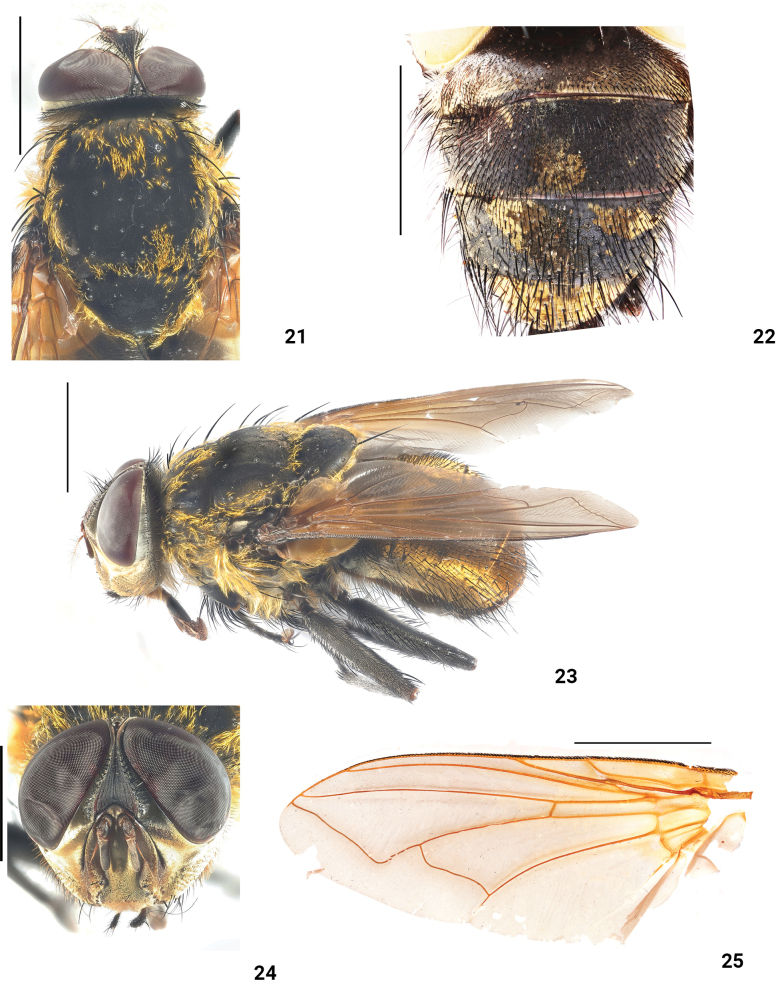
*Xanthotryxushuapingensis* Liu, Li & Yao, sp. nov. male (holotype) **21** head, thorax, dorsal view **22** abdomen, dorsal view **23** habitus, lateral view **24** head, anterior view **25** wing. Scale bars: 3 mm.

***Head*** (Figs 21, 23, 24). Eyes red, bare; frons black, with sparse short black hair, eminence near antennae; interfrontalia surface linear at narrowest part; parafacial and mediane dull red, bare, with yellowish-white tomentum; lunule bare; antennae brownish, with yellowish-white tomentum, third antennal segment about 2.5 times longer than second antennal segment, arista plumose; facial carina well developed; third antennal segment as long as distance from vibrissa to epistoma; gena black, with dense black hairs; eyes 3 times higher gena; palpus black.

***Thorax*** (Figs 21, 23). black, with dense crinkly golden hairs; acr 2+3, dc 2+3, ial 0+2, h 4, ph 3+0, pra 1, sal 2, pal 2; anterior and posterior spiracles black, proepimeral bristles present; anepisternum with a dense tuft of crinkly golden hairs on the posterior margin, mpl 0+6, spl 1+1, ppl 3, with a tuft of crinkly golden hairs; postalar declivity with a dense tuft of crinkly golden hairs.

***Wing*** (Fig. 25) brownish-hyaline; epaulet and basicostal scale black; subcostal sclerite yellowish, with dense yellow tomentum and 2–3 setae; radial stem vein bare, radial vein knob with yellow tomentum; upper calypter and lower calypter reddish-brown.

***Legs*** (Fig. 23) black, femora with white tomentum; tarsi with dense short yellow hairs on ventral surface; fore femur with 11 d, 13 pd, 14 v; mid femur with 11 v; mid tibia with 1 ad, 2 pd; hind femora with 1 d, 14 ad, 1 pd, 10 v, 7 av; hind tibia with 3 ad, 5 pd.

**Figures 26–30.**
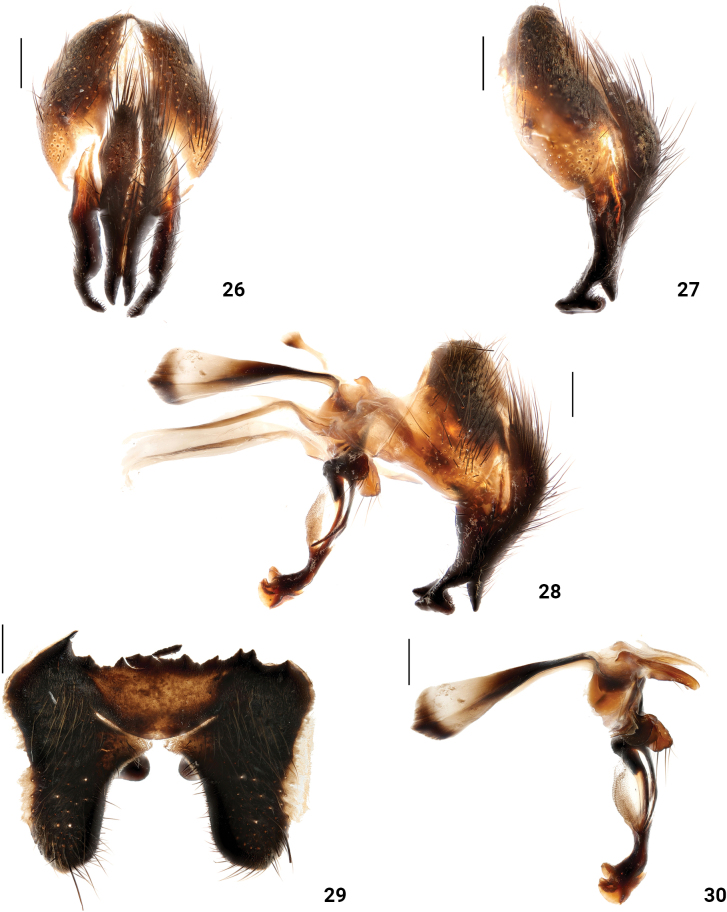
*Xanthotryxushuapingensis* Liu, Li & Yao, sp. nov. male terminalia **26** cerci and surstyli, posterior view **27** cerci and surstyli, lateral view **28** terminalia lateral view **29**ST_5_ ventral view **30** phallic complex, lateral view. Scale bars: 0.3 mm.

***Abdomen*** (Figs 22, 23) black, tessellate yellow sarcophagids markings; ST_1_ with yellow setae, ST_2–5_ with black setae and yellow setae; ST_5_ two prominences on the ventral surface (Fig. 29). Male terminalia: end of surstyli curved inward in posterior view (Fig. 26); end of cerci sharp and curved backward in posterior view (Fig. 26); surstyli hook-like in lateral view (Figs 27, 28); pregonites with a row of black setae, postgonites bare; paraphallus slender and curved forward; hypophallus membranous; acrophallus well developed and terminal trumpet (Figs 28, 30).

**Female.** Unknown.

##### Measurements.

Male. Body length 11.3–14.5 mm.

##### Etymology.

The specific epithet is chosen after Huaping National Nature Reserve, Guangxi Province where the holotype was collected.

##### Distribution.

China (Guangxi).

##### Remarks.

The new species is similar to *X.draco* Aldrich, 1930, but it differs by the following points: h 4, ph 3+0, ppl 3, base of the antennae distinctly separated, terminal extension of surstyli hook-like in lateral view. The new species paraphallus is slender, and the acrophallus is not as developed as in *X.draco*. The new species phallus is rather similar to that of *X.mongol* and *X.uniapicalis*, but can be separated from the latter two as follows: the end of the *X.mongol* paraphallus is approximately angular curved, while in the new species the paraphallus terminal is arc-shaped and bent; moreover, the new species paraphallus is thinner than that of *X.mongol*; the new species paraphallus does not bend forward beyond the hypophallus in lateral view, whereas the *X.uniapicalis* paraphallus bends forward over the hypophallus in lateral view.

## Supplementary Material

XML Treatment for Dexopollenia

XML Treatment for Dexopollenia guangxiensis

XML Treatment for Dexopollenia chongqingensis

XML Treatment for Xanthotryxus

XML Treatment for Xanthotryxus huapingensis

## ﻿Additional information

### Author ORCIDs

Shixin Liu https://orcid.org/0009-0007-7102-9061

Honghu Ji https://orcid.org/0009-0001-8217-7293

Wenliang Li https://orcid.org/0000-0001-9019-1223

Gang Yao https://orcid.org/0000-0002-3551-5878

### Data availability

All of the data that support the findings of this study are available in the main text.
